# Predictors of loss to follow-up among people living with HIV on antiretroviral therapy in a rural health facility using paper-based records

**DOI:** 10.3389/fpubh.2025.1623805

**Published:** 2025-08-21

**Authors:** Michael Abugah, Angela Mwinorme Yabelang, John Kobla Akorlie, Haruna Mahama, Benjamin Abuaku

**Affiliations:** ^1^Department of Epidemiology and Disease Control, School of Public Health, College of Health Sciences, University of Ghana, Accra, Ghana; ^2^St. Theresa’s Hospital, Nandom, Ghana; ^3^Epidemiology Department, Noguchi Memorial Institute for Medical Research, College of Health Sciences, University of Ghana, Accra, Ghana

**Keywords:** LTFU, HIV/AIDS, rural health facility, ART, PLWHIV, Ghana

## Abstract

**Introduction:**

Most studies on loss to follow-up (LTFU) among people living with HIV are done in urban Antiretroviral Therapy (ART) centers that have electronic medical records system. However, there are limited studies in ART centers in rural areas that rely solely on paper-based medical records (PBMR). This study aimed to determine the incidence, trends, and predictors of LTFU among people living with HIV at a rural health facility in Ghana that rely on PBMR.

**Methods:**

A retrospective cohort analysis of 232 HIV registrants who received care at St. Theresa’s Hospital, Nandom Municipality, Ghana between 2018 and 2022 was conducted. The Kaplan–Meier method was used to determine failure probabilities, and the Cox proportional hazard regression was used to identify predictors of LTFU.

**Results:**

The incidence proportion of LTFU was 24.14%, with a rate of 9.57 per 1,000 p-m. There was a significant decline in cases of LTFU from 2018 to 2022, although registrants under 25 years and males exhibited an increase in LTFU risk from 2021 to 2022. Registrants who had a viral load of 1,000 copies or more had an increased risk of LTFU (aHR = 3.52, 95% CI: 1.39–9.00). Conversely, adherence to ART (aHR = 0.28, 95% CI: 0.12–0.68), HIV status disclosure (aHR = 0.34, 95% CI: 0.14–0.84), and being in WHO stage 2 (aHR = 0.10, 95% CI: 0.03–0.31) or stage 3 (aHR = 0.21, 95% CI: 0.08–0.52) acted as protective factors for LTFU.

**Conclusion:**

This study identified key predictors of LTFU among people living with HIV in a rural health facility, providing valuable insights to the existing literature. Targeted strategies should prioritize viral suppression, support ART adherence, and encourage status disclosure to improve retention, particularly in rural settings.

## Introduction

Human immunodeficiency virus (HIV) infection remains a global threat despite significant progress in its diagnosis, treatment, and methods of prevention. At the end of 2022, the global burden of HIV was 39 million, with 1.3 million new infections (1). Africa accounts for two-thirds of global HIV infections (2), with 1 in every 25 adults living with the disease (2, 3). In Ghana, the national HIV prevalence is 1.68%, with 346,120 people living with the infection (4, 5). In the Upper West Region of Ghana, the prevalence of HIV is 1.65%, with over 5,000 people living with the disease (5).

Antiretroviral Therapy (ART) has significantly enhanced the well-being and life expectancy of people living with HIV infection (PLHIV) (6). In the past, surviving for more than 10 years with the disease was unlikely (7). However, today, due to the global expansion of ART access and coverage, individuals with HIV can expect to live long and healthy lives (6, 8). Since its introduction, ART has played a vital role in averting HIV-related deaths. According to a recent report by the Joint United Nations Programme on HIV/AIDS, ART has saved 21 million lives (9). Also, from 1995 to 2015, ART prevented 9.5 million global deaths (10). If the global targets set by the Joint United Nations Program on HIV/AIDS are achieved through increased ART treatment availability, a projected 34.9 million deaths and 40.2 million new HIV infections could be prevented (10). ART is highly effective in suppressing viral load, reducing the risk of transmission, and improving survival rates for HIV-positive individuals (10–12).

Acknowledging the significance of ART in the battle against the HIV pandemic, it is unfortunate that ART does not provide a cure for HIV infection; rather, it suppresses the viral load, necessitating regular intake of antiretroviral drugs throughout a patients’ lifetimes. Continuous uptake of ART is crucial for achieving the UNAIDS 95-95-95 targets. However, continuous uptake of ART is heavily reliant on patient retention in care (13). Patient retention in care serves various critical purposes such as initiating ART, monitoring medication side effects, ensuring continuous access to medication, diagnosing treatment failure, and changing to second or third-line ART regimens if needed (14). This help maintain high medication adherence, achieve viral suppression, improve health outcomes, and minimize the risk of horizontal transmission (6, 10, 11, 14). Nonetheless, retaining patients in HIV treatment for a prolonged period can be challenging, especially in low- and middle-income countries like Ghana (15, 16). Interventions, such as patient education, counseling and support, the use of peer navigators and community health workers, and the integration of HIV care with other health services, have been implemented to improve patient retention (17, 18). Despite these interventions, several low-setting facilities in sub-Saharan Africa; still face challenges in retaining HIV patients in care (18, 19).

LTFU characterizes situations where HIV patients on antiretroviral therapy (ART) disengage from care without prior notification of transfer, death, or cessation of ART. However, the specific duration of this disengagement varies. Different studies have adopted varying timeframes to define LTFU, but all use the last review date as a reference point. Some studies done in different settings has defined LTFU as an absence from care for one (20), three (21), six (22), or even 12 months (23). Moreover, some studies have reported more longer periods, such as 180 months (24, 25). Various studies done in Tanzania, Congo, Brazil, Ethiopia, Zimbabwe, and 46 other African countries has reported incidence proportion of LTFU ranging from as low as 1.6% to as high as 36% (26–31). In Ghana, a study by Sifa et al. (16) defined loss to follow-up as occurring when an HIV registrant failed to visit the clinic more than 90 days after their scheduled appointment and had not been recorded as ‘deceased’ or ‘transferred out’ in the database. The incidence proportion of LTFU in an urban ART Center in Ghana has been reported to be 26.9% (16), surpassing the WHO’s recommended LTFU target rate of less than 15% (32).

Notably, most of the studies done on LTFU primarily focus on major hospitals in urban settings where access to electronic patient records is prevalent. However, the scarcity of research in deprived rural areas, such as the Nandom district, where sole reliance on paper-based medical records persists, poses a gap in determining the extent of LTFU in such settings. In the context of the Nandom district, no formal study has been conducted to determine the incidence of LTFU. This lack of local insight hampers the implementation of targeted interventions essential for effectively retaining HIV patients in care within the district.

Several factors have been associated with LTFU which can be categorized into: patient-related factors such as age, body mass index, and clinical stage of disease at presentation (33, 34); treatment-related factors such as poor adherence, comorbid with tuberculosis, isoniazid prophylaxis, ART side effects, changing ART, duration on ART, viral load, CD4 count, WHO stages III &IV, being bed-ridden, and ambulatory patient (33); healthcare system-related factors, and socio-economic factors (35). LTFU can lead to poor health outcomes for patients, reduce the effectiveness of HIV treatment and care programs, increase transmission in the community, and increase healthcare costs (13). Patients who are lost to follow-up have an elevated risk of opportunistic infections and death (36). LTFU is also associated with lower CD4 counts, less effective ART, and drug-resistant HIV risk (37). LTFU has been shown to increase HIV transmission and reduce testing and treatment uptake in communities (38, 39). HIV LTFU also has negative consequences for health systems, including increased costs, reduced efficiency, and decreased quality of Mugglin et al. and Nglazi et al. (38, 40). LTFU has negative consequences for countries and the global response to the HIV epidemic, including reduced progress toward achieving the UNAIDS 95–95-95 targets and increased healthcare costs (15, 41).

Knowing the trends and predictors of LTFU among HIV patients in the Nandom District will help initiate interventions that are appropriate and locally suited for the district to ensure the retention of HIV patients in care. Therefore, this study aimed to determine the incidence, trends, and predictors of loss to follow-up among HIV registrants in the Nandom district.

## Materials and methods

### Study design and setting

**Figure 1:**
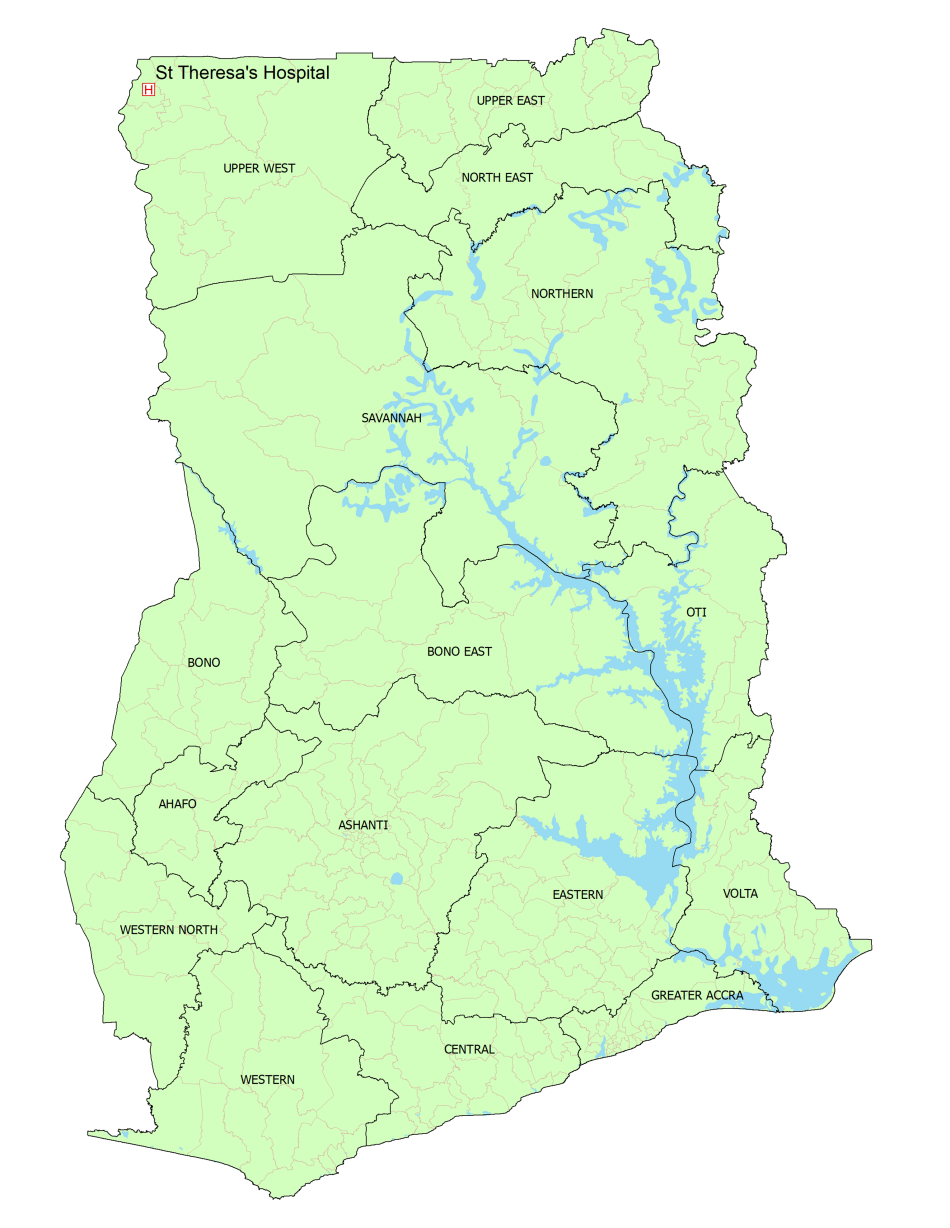
Map of Ghana showing the 16 administrative regions and study site (St. Theresa’s Hospital).

This was a hospital-based retrospective cohort study conducted in the St Theresa’s Hospital using patient records covering 1st January 2018 to 31st December 2022. The hospital is found in Nandom Municipality which is in the Upper West Region of Ghana (Figure 1). It shares border with Hamile and Burkina Faso to the north, Lawra district to the south, Lambussie district to the east, and Black Volta to the west. The facility acts as a referral hospital within the Nandom municipality and serves neighboring districts (Lambussie, Sissala west) and patients from Burkina Faso. The facility serves a population of 51,328 (42). It is also the only ART Center within the municipality. The ART clinic was established in 2005 and currently renders ART care to 490 patients (5).

### Sample size determination and sampling method

The study used sample size calculation for estimating a single population proportion (43) to determine the required minimum sample. A retrospective cohort study done in the Greater Accra regional hospital, Ghana, reported incidence proportion of LTFU among HIV registrants as 26.9% (16). Based on this reported incidence proportion in a predominantly urban population in Ghana, we hypothesized that the incidence proportion of LTFU among the HIV registrants in the St Theresa’s hospital, predominantly rural population, will be lower than 26.9% at 95% confidence interval and 5% margin of error. Incomplete medical records of the cohort that were excluded from the study were 6.07%. Adjusting for this, the overall minimum sample size required for the study was 228.

### Study population and variables

The study population were all HIV Registrants who initiated ART treatment at the St Theresa’s hospital from 1st January 2018 to 31st December 2022. Those who had unknown ART initiation date and incomplete baseline data were excluded from the study. Transfers into the hospital were included only if their ART initiation date and baseline data were fully documented. Transfers out of the facility were treated as censored observations in the analyses. Patients who re-started ART after treatment interruption were included only if complete baseline data at re-initiation were available; otherwise, they were excluded. The primary outcome variable of interest was Loss to Follow-up (LTFU) which was defined in this study as failure of an HIV Registrant to attend scheduled medical appointment or pick up medications for a 90-day period (3 months) starting from the last date of reporting to the hospital without any documented transfer, death, or cessation of ART. The predictor variables assessed were baseline socio-demographic, disease-related, and treatment-related factors. The sociodemographic factors assessed were sex, age, employment status, marital status, educational level, mobility status (walk without support or with support), availability of recorded personal contact number, disclosure status (patient informing at least one individual of his/her HIV positive status), and availability of caregiver support. The disease-related factors assessed were type of virus, WHO defined stage of infection, comorbidity with tuberculosis, and viral load. Treatment-related factors assessed were type of ART combination, use of co-trimoxazole as prophylaxis, change of initial ART combination, and ART adherence status.

### Data collection procedures

Data extraction was done from 1st September 2023 to 31st October 2023. A structured data extraction tool, developed based on study objectives and relevant literature, was used to manually extract data from patients’ paper-based medical folders, laboratory result reports, and attendance registers. Two (2) independent data extractors and two (2) HIV-care nurses performed the manual extraction. Each day, the supervisor (a health information officer) and principal investigator reviewed the extracted data to identify and resolve any discrepancies thereby ensuring consistency and completeness throughout the extraction period. All extracted data were anonymized by removing patient identifiers prior to analysis. Data extractors and the supervisor were trained on confidentiality, and data were securely stored in a password-protected electronic files accessible only to authorized study personnel.

### Data analysis

All variables were described using means, medians, and proportions depending on type of variable and its uniformity. All estimates were presented with their respective 95% confidence intervals, standard deviation, or interquartile range. Kaplan Meier survival analysis was used to estimate the incidence of LTFU and the failure probabilities at time 0, 12, 24, 36, 48, and 60 months. Univariate cox proportional hazard regression model was used to describe hazard ratio of participants lost to follow-up with associated 95% confidence interval (CI). All variables with *p*-values <0.05 were considered statistically significant and were fitted into multivariable cox proportional hazard regression model. Proportional-hazard assumption test based on Schoenfeld residuals (phtest) was conducted to assess the validity of the model (Supplementary Figure S1). All statistical analysis were done using STATA 17 BE version.

## Results

### Socio-demographic characteristics

The study population consisted of 232 HIV Registrants with median age of 37 years (IQR: 31, 46). Majority of participants were females (61.6%), married (54.3%), employed (79.7%), and had formal education (63.4%). Most participants walked without support (84.8%), had a contact number in their folder (80.2%), had disclosed their HIV status (82.3%), and had caregiver support (84.9%) (Table 1).

**Table 1:** Socio-demographic characteristics of study participants.

Characteristics	Frequency (*N* = 232)	Prop (%)	95% CI of Prop.
Median Age in years (IQR): 37.0 (31.0–46.0)			
Age group (years)			
<25	27	11.6	8.1–16.5
25–49	162	69.8	63.6–75.4
≥50	43	18.5	14.0–24.1
Sex			
Male	89	38.4	32.3–44.8
Female	143	61.6	55.2–67.7
Marital Status			
Single	55	23.7	18.7–29.6
Married	126	54.3	47.8–60.7
Separated/Widow	51	22.0	17.1–27.8
Employment Status			
Employed	185	79.7	74.1–84.5
Unemployed	47	20.3	15.6–26.0
Educational Status			
No formal education	85	36.6	30.7–43.1
Basic	54	23.3	18.3–29.2
Junior High School	47	20.3	15.6–26.0
Senior High School	25	10.8	7.4–15.5
Tertiary	21	9.1	6.0–13.5
Mobility Status			
Walk without support	196	84.5	79.2–88.6
Walk with support	36	15.5	11.4–20.8
Availability of recorded personal contact number			
No	46	19.8	15.2–25.5
Yes	186	80.2	74.5–84.8
Disclosure status			
HIV status not disclosed	41	17.7	13.3–23.2
HIV status disclosed	191	82.3	76.8–86.7
Availability of caregiver support			
No	35	15.1	11.0–20.3
Yes	197	84.9	79.7–89.0

HIV, Human Immunodeficiency Virus; CI, Confidence Interval; Prop, proportion, IQR, interquartile range.

### Disease-related and treatment-related characteristics

All the participants had infection with HIV type 1 virus (100%). Majority of participants were in WHO defined stage 2 of the infection (36.21%), had no comorbidity with TB (89.7%) and had a viral load of <1,000 copies/mL (93.1%) (Table 2). The median viral load was 40 copies/mL [IQR: 0–80]. Regarding the year of initiation of ART, 19.4% started in 2018, 18.5% in 2019, 22.0% in 2020, 18.1% in 2021, and 22.0% in 2022. Most participants were on either TDF + 3TC + DTG (48.7%) or TDF + 3TC + EFV (42.2%), while a few (9.1%) were on other types of ART regimen (TDF + 3TC + NVP, AZT + 3TC + NVP, AZT + 3TC + EFV, ABC + 3TC + EFV, CBV + EFV). Majority of participants were kept on co-trimoxazole prophylaxis (82.3%), did not change initial ART (84.5%), and adhered to ART (82.3%) (Table 2).

**Table 2:** Disease-related and treatment-related characteristics of study participants.

Characteristics	Frequency (*N* = 232)	Prop (%)	95% CI
Type of HIV			
Type 1	232	100.0	
Type 2	0	0.0	
WHO defined stage of HIV infection			
Stage 1	68	29.3	23.8–35.5
Stage 2	84	36.2	30.3–42.6
Stage 3	69	29.7	24.2–36.0
Stage 4	11	4.8	2.6–8.4
Comorbidity with TB			
No	210	90.5	86.0–93.7
Yes	22	9.5	6.3–14.0
Viral Load			
<1,000 copies/mL	216	93.1	89.0–95.7
≥1,000 copies/mL	16	6.9	4.3–11.0
Year of ART initiation			
2018	45	19.4	14.8–25.0
2019	43	18.5	14.0–24.1
2020	51	22.0	17.1–27.8
2021	42	18.1	13.6–23.6
2022	51	22.0	17.1–27.8
Type of art regimen combination			
TDF + 3TC + DTG	113	48.7	42.2–55.2
TDF + 3TC + EFV	98	42.2	36.0–48.7
Others^¥^	21	9.1	6.0–13.5
Co-trimoxazole prophylaxis			
No	41	17.7	13.3–23.2
Yes	191	82.3	76.8–86.7
Change in ART			
No	196	84.5	79.2–88.6
Yes	36	15.5	11.4–20.8
Adherence to ART			
No	41	17.7	13.3–23.2
Yes	191	82.3	76.8–86.7

WHO, world health organization; HIV, human immunodeficiency virus; TB, tuberculosis; ART, antiretroviral therapy; TDF, Tenofovir Disoproxil Fumarate; 3TC, Lamivudine; DTG, Dolutegravir; EFV, Efavirenz; NVP, Nevirapine; AZT, Zidovudine; ABC, Abacavir; CBV, Combivir; Prop, proportion; CI, Confidence Interval. ^¥^TDF + 3TC + NVP (*n* = 5); AZT + 3TC + NVP (*n* = 1); AZT + 3TC + EFV (*n* = 8); ABC + 3TC + EFV (*n* = 5); CBV + EFV (*n* = 2).

### Follow-up outcomes

**Figure 2:**
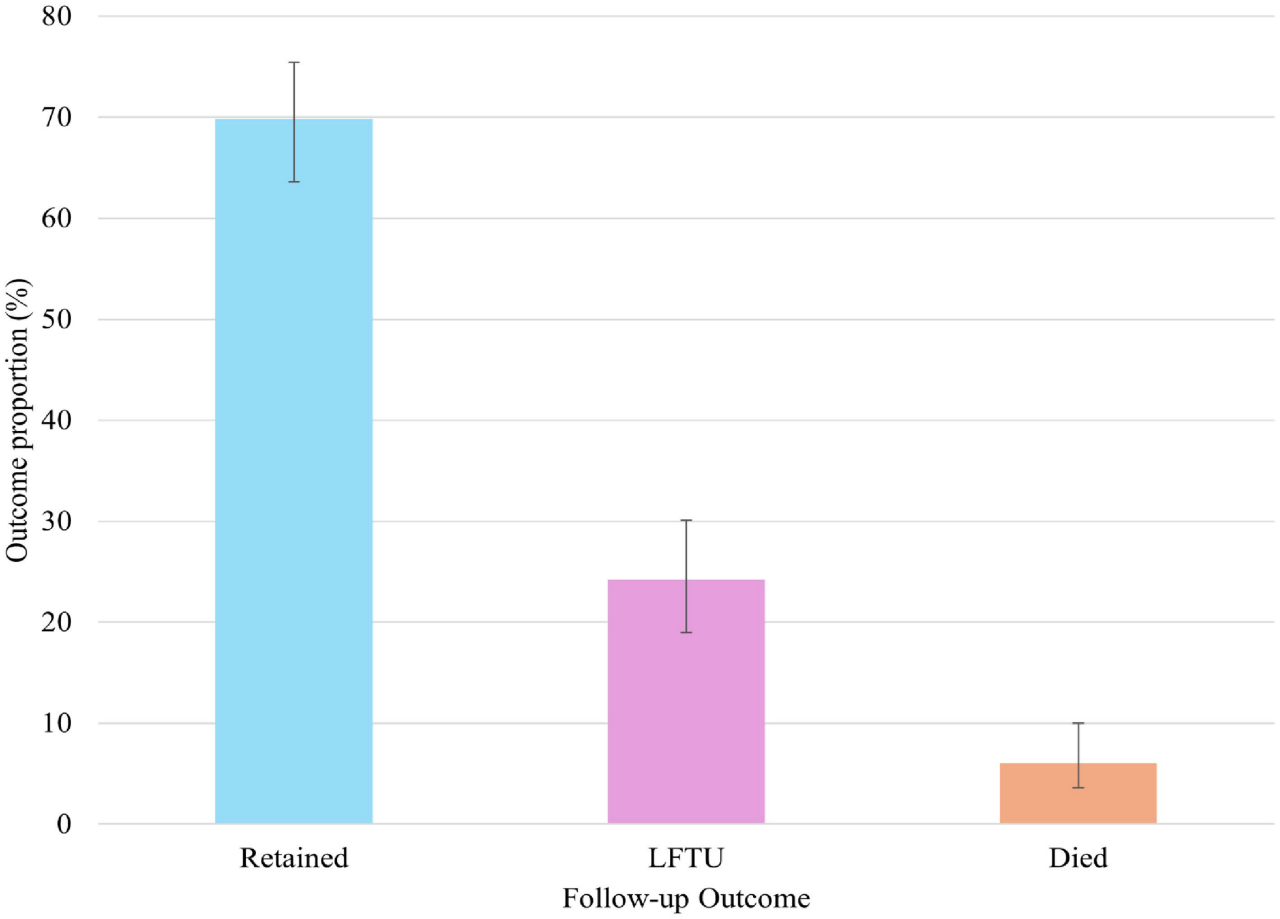
Follow-up outcomes of HIV registrants from 2018 to 2022.

At the end of the 5 years follow-up, 69.8% (95% CI, 63.6–75.4) were retained in care, 24.2% (95% CI, 19.0–30.1) were lost to follow-up, and 6.0% died (95% CI, 3.6–10.0) (Figure 2).

### Incidence proportion

**Figure 3:**
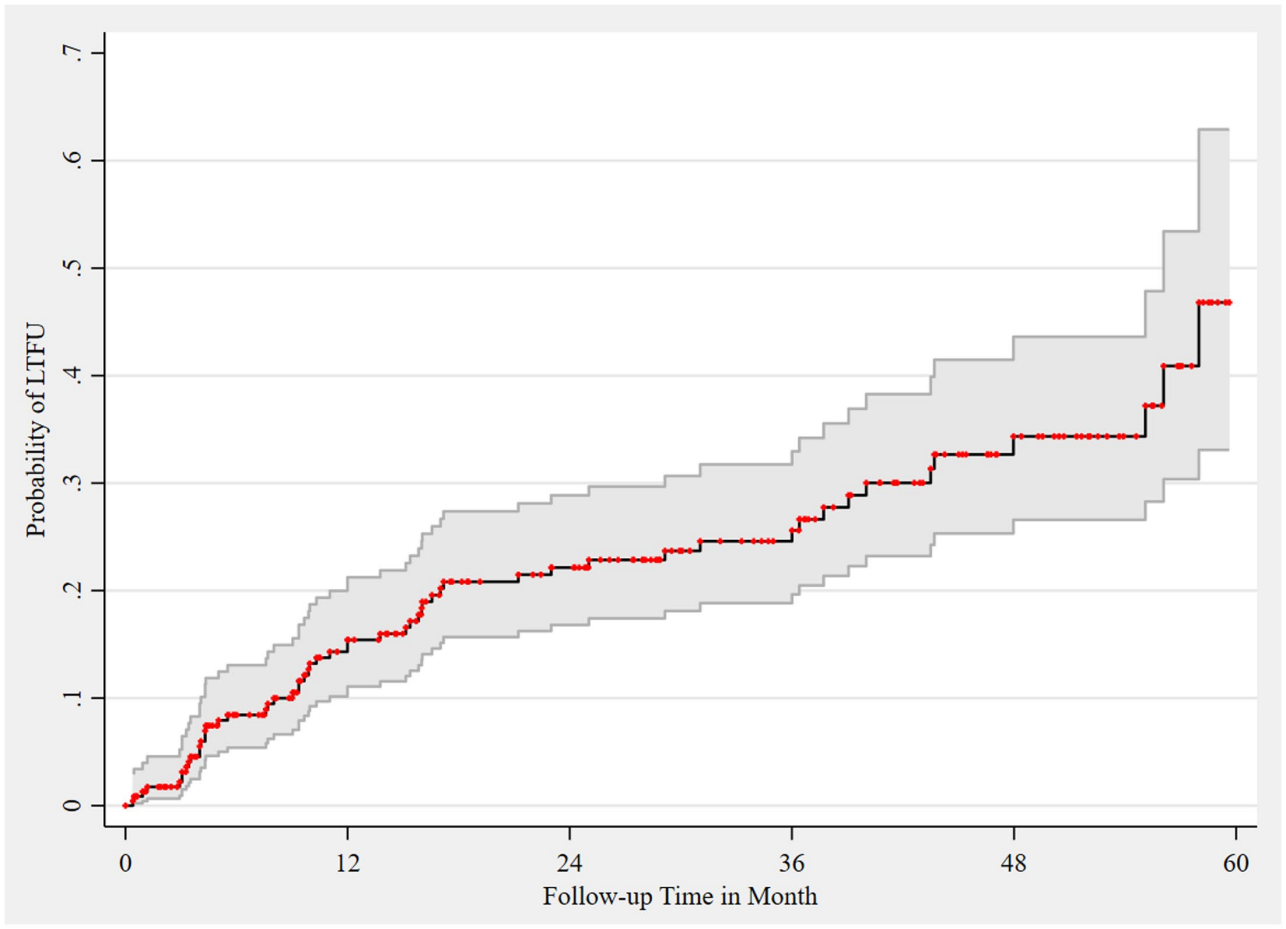
Failure estimate of LTFU among HIV registrants, 2018–2022. Area shaded gray represents 95% CI of failure estimates, which are represented as red dots.

The proportion of LTFU in the cohort was 24.14% (95% CI, 19.04–30.09). The probability of LTFU among the HIV registrants was 15.4, 22.2, 24.6, 34.4, and 46.8% at 12, 24, 36, 48, and 60 months of follow-up, respectively (Figure 3).

### Incidence rate of LTFU

**Figure 4:**
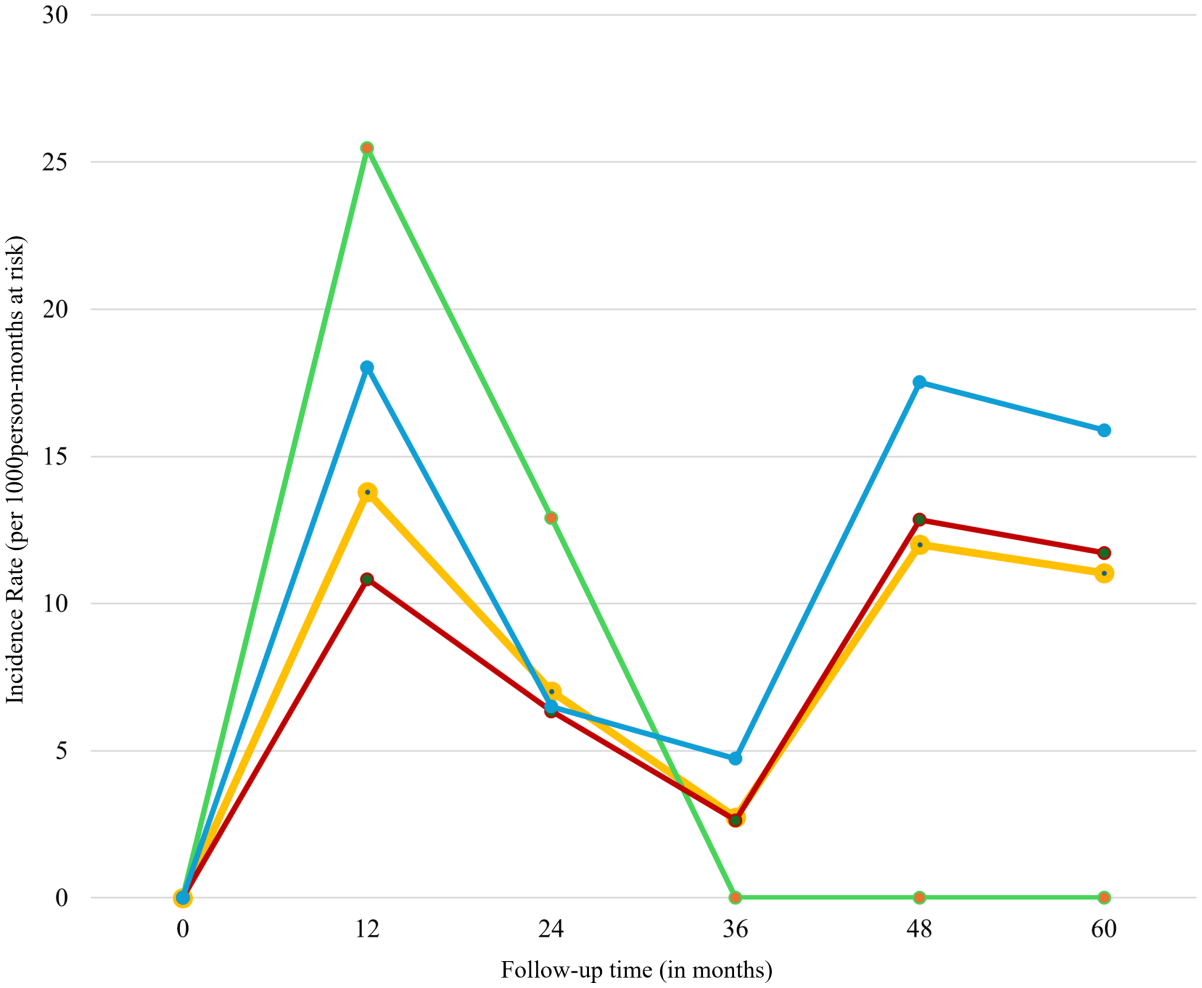
Incidence rate of LTFU of HIV registrants by follow up time, 2018–2022. Green line represents registrants less than 25 years; red line represents registrants aged 25–49 years; blue line represents registrants aged 50 years and above; and yellow line represents all age-groups.

We followed the registrants for a median of 23.62 months (IQR: 7.85–41.54) after the initiation of antiretroviral therapy (ART). During this period, we observed 56 cases of loss to follow-up (LTFU) over a total of 5850.05 person-months (p-m) at risk. The overall incidence rate of LTFU was 9.57 per 1,000 p-m (95% CI: 7.37–12.44). The risk of LTFU, both overall and age-specific (≤24 years, 25–49 years, and ≥50 years), was highest in the first 12 months of follow-up. This was followed by a significant decrease in risk up to 36 months, a gradual increase at 48 months except for age group ≤ 24 years, and then a decline at 60 months of follow-up (Figure 4).

### Trend of LTFU

**Figure 5:**
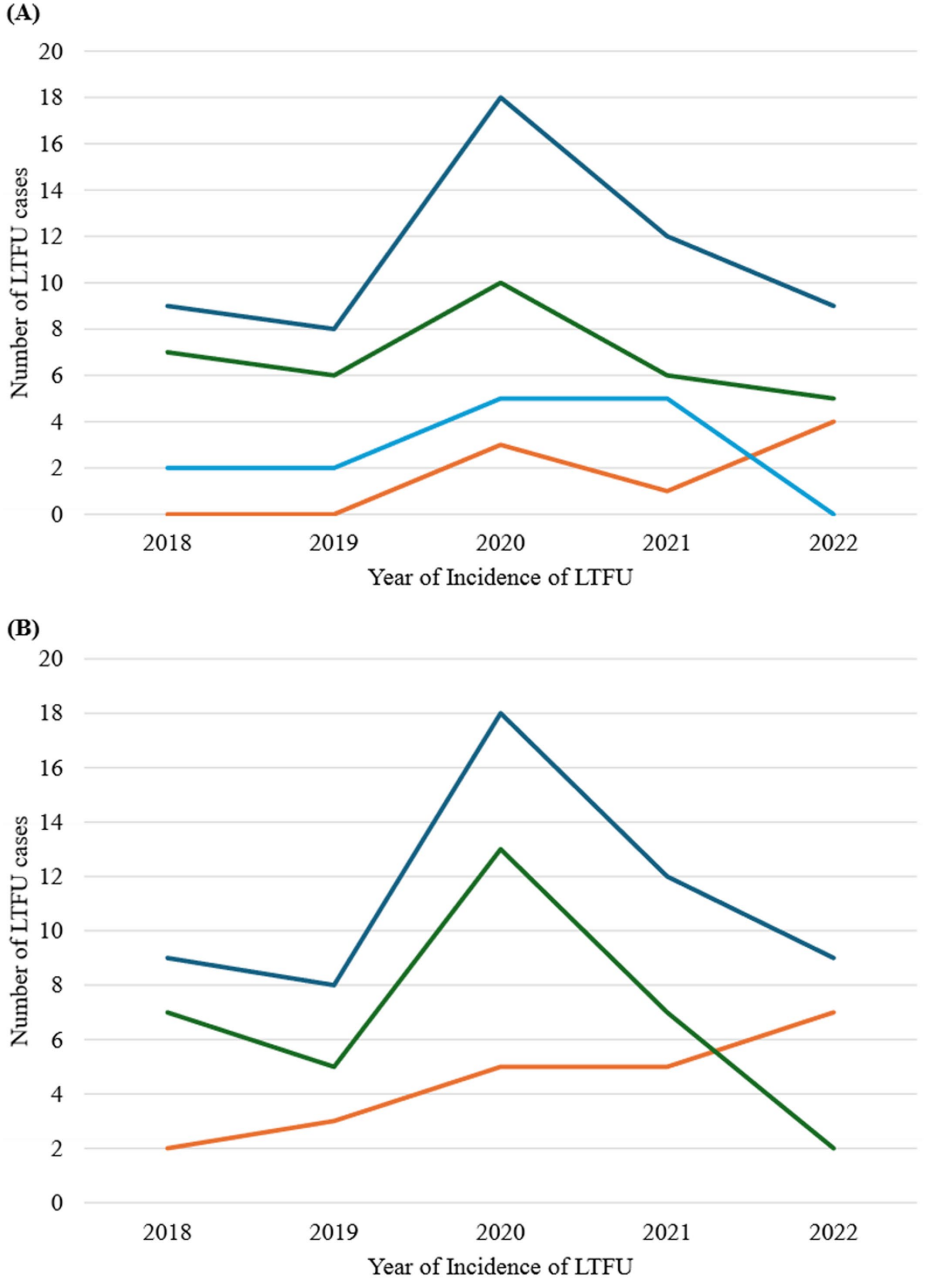
Trend analysis of LTFU stratified by age groups and sex of registrants, 2018–2022. **(A)** Stratification by age group. Orange line represents registrants aged less than 25 years; green line represents registrants aged 25–49 years; light blue line represents registrants aged 50 years and above; and deep blue represents all age-group. **(B)** Stratification by sex. Orange line represents males; green line represents females; and blue line represents both sexes.

Figure 5 presents the trend analysis of loss to follow-up (LTFU) cases stratified by age groups and sex of registrants. Overall, LTFU cases were low from 2018 to 2019, followed by a significant increase from 2019 to 2020, and then a decline from 2021 to 2022. This trend was consistent across all three age groups (≤24 years, 25–49 years, and ≥50 years) and both sexes, except for registrants ≤ 24 years (Figure 5A) and males (Figure 5B), who exhibited an upward trajectory from 2021 to 2022.

### Predictors of LTFU among HIV registrants

In a univariable cox proportional hazard regression analysis, the predictors of LTFU included mobility status, availability of recorded personal contact number, disclosure status, availability of caregiver support, WHO defined stage of HIV infection, comorbidity with Tuberculosis, viral load, year of ART initiation, type of ART regimen combination, co-trimoxazole prophylaxis, change of initial ART, and adherence to ART (Table 3). However, after adjusting for all these significant predictors in a multivariable cox proportional hazard regression analysis, the predictors that remain statistically significant were disclosure status, WHO defined stage of HIV infection, viral load, year of ART initiation, and adherence of ART.

**Table 3:** Predictors of LTFU among HIV registrants.

Predictor variables	HR	95% CI	*p-*value	aHR	95% CI	*p-*value
Age	1.00	0.98–1.02	0.942			
Age group						
<25^*^						
25–49	0.67	0.31–1.46	0.316			
≥50	0.98	0.41–2.35	0.971			
Sex						
Male^*^						
Female	0.88	0.52–1.51	0.650			
Marital status						
Single^*^						
Married	0.72	0.39–1.35	0.310			
Separated/widow	0.72	0.34–1.54	0.396			
Employment status						
Employed^*^						
Unemployed	1.07	0.57–2.03	0.831			
Education						
No formal education^*^						
Formal education^§^	1.04	0.61–1.79	0.878			
Mobility status						
Walk without support^*^						
Walk with support	3.35	1.87–6.00	<0.001	1.47	0.71–3.05	0.295
Availability of recorded personal contact number						
No^*^						
Yes	0.10	0.06–0.18	<0.001	1.04	0.42–2.56	0.295
Disclosure status						
HIV status not disclosed^*^						
HIV status disclosed	0.09	0.05–0.15	<0.001	0.34	0.14–0.84	0.020
Availability of Caregiver support						
No^*^						
Yes	0.08	0.05–0.14	<0.001	0.63	0.25–1.59	0.329
WHO defined stage of HIV infection						
Stage 1^*^						
Stage 2	0.05	0.02–0.14	<0.001	0.10	0.03–0.31	<0.001
Stage 3	0.11	0.05–0.24	<0.001	0.21	0.08–0.52	0.001
Stage 4	0.15	0.04–0.61	0.008	0.42	0.08–2.13	0.295
Comorbidity with Tuberculosis						
No^*^						
Yes	4.28	2.35–7.79	<0.001	2.12	1.00–4.51	0.052
Viral load						
<1,000 copies^*^						
≥1,000 copies	3.54	1.82–6.88	<0.001	3.52	1.39–9.00	0.008
Year of ART initiation						
2018^*^						
2019	2.64	0.85–8.23	0.095	3.73	0.97–14.25	0.055
2020	11.64	3.41–39.69	<0.001	24.55	4.46–45.12	<0.001
2021	14.45	3.94–53.00	<0.001	21.79	3.84–48.66	0.001
2022	37.55	9.15–154.14	<0.001	28.75	4.39–58.83	<0.001
Type of ART regimen combination						
TDF + 3TC + DTG^*^						
TDF + 3TC + EFV	0.35	0.18–0.62	0.001	1.43	0.50–4.12	0.506
Others^¥^	0.41	0.15–1.10	0.077	1.55	0.41–5.86	0.517
Co-trimoxazole prophylaxis						
No^*^						
Yes	0.08	0.04–0.13	<0.001	0.46	0.18–1.17	0.102
Change of ART						
No^*^						
Yes	6.81	4.02–11.54	<0.001	0.58	0.24–1.36	0.207
Adherence of ART						
No^*^						
Yes	0.08	0.05–0.14	<0.001	0.28	0.12–0.68	0.005

^*^Reference group. WHO, world health organization; HIV, human immunodeficiency virus; ART, antiretroviral therapy; TDF, Tenofovir Disoproxil Fumarate; 3TC, Lamivudine; DTG, Dolutegravir; EFV, Efavirenz; NVP, Nevirapine; AZT, Zidovudine; ABC, Abacavir; CBV, Combivir; CI, confidence interval. ^¥^TDF + 3TC + NVP, AZT + 3TC + NVP, AZT + 3TC + EFV, ABC + 3TC + EFV, CBV + EFV. ^§^Basic school, Junior High School, Senior High School, and Tertiary.

Registrants who disclosed their HIV status had a lower risk of LTFU compared to those who did not disclose their status (aHR-0.34, 95% CI: 0.14–0.84). Registrants who were classified as WHO defined stage 2 (aHR-0.10, 95% CI: 0.03–0.31) and stage 3 (aHR-0.21, 95% CI: 0.08–0.52) had reduced risk of LTFU compared to those who were in stage 1. Also, registrants whose viral load were 1,000 copies or more had increased risk of LTFU compared to those who had viral loads less than 1,000 copies (aHR-3.52, 95% CI: 1.39–9.00). Moreover, the risk of LTFU increased among registrants who initiated ART in the year 2020 (aHR-24.55, 95% CI: 4.46–45.12), 2021 (aHR-21.79, 95% CI: 3.84–48.66), and 2022 (aHR-28.75, 95% CI: 4.39–58.83) as compared to those who initiated in the year 2018. Additionally, registrants who adhered to ART had a lower risk of LTFU than those who did not adhere (aHR-0.28, 95% CI: 0.12–0.68).

## Discussion

This study determined the incidence, trends, and predictors of loss to follow-up (LTFU) among HIV registrants on antiretroviral therapy (ART) in a rural hospital which relied on paper-based medical records. Although it has been reported that paper-based records may be prone to incomplete documentation, missing data and inconsistencies (44), the records at our study site were detailed and well-maintained with a high degree of completeness (94%). This finding has also been documented in other settings where paper-based medical records were found to have higher completeness levels and content quality compared with electronic medical records (45, 46). This may be a demonstration of the fact that despite the inherent limitations of paper-based records, rural health facilities without electronic systems can still generate high-quality data to inform policy. The main challenge encountered was the time-consuming nature of manual data collection and the need for cross-validation to ensure accuracy. This experience underscores the importance of transitioning to electronic medical record systems to improve efficiency and streamline data management in similar settings.

The incidence proportion of LTFU in this study was 24.14%, which falls within the reported range of 1.6 to 36% in Sub-Saharan Africa (29). Our observed incidence is higher than the 19.21% reported in Ethiopia (41) but lower than the 26.9% reported in Accra, Ghana (16), and the 25.1% reported in South Africa (44). These differences could be attributed to variations in study duration, geographical locations, and study designs. Despite the lower rate of LTFU in our study compared to those in Accra and South Africa, it is crucial to retain all patients in treatment, as LTFU is associated with adverse health outcomes, including increased HIV transmission and mortality (36).

Our observed incidence rate of LTFU of 9.57 per 1000p-m aligns closely with the rate of 10.90 per 100 person-years (equivalent to 9.08 per 1,000 person-months) reported in Ethiopia (41). However, our rate is higher than the 3.7 per 100 person-years (equivalent to 3.08 per 1000p-m) reported by Birhanu et al. (45) and the 7.5 per 100 person-years (equivalent to 6.25 per 1000p-m) observed by Kiwanuka et al. (33). Conversely, our reported rate is lower than the rate of 21 per 1000pn-m reported by Opio et al. (19). These disparities in rates could be attributed to variations in study settings. Our study was conducted in a rural setting, whereas both the studies by Birhanu et al. (45) and Kiwanuka et al. (33) were conducted in urban settings. It has been reported that rural residents have a higher risk of LTFU compared to urban residents (47), which may explain the higher rates observed in our study compared to these urban-based studies. Also, our study was conducted in the Nandom district of the Upper West Region of Ghana, where the HIV prevalence is relatively low at 1.65% (5). In contrast, Opio et al. (19) conducted their study in Wakiso district, Uganda, which has a higher HIV prevalence of 10%. Furthermore, our study was carried out in the sole ART center in the Nandom district, whereas Opio et al. conducted their work across multiple facilities including a hospital and several health centers in Wakiso district (19). This difference in study settings suggests that while some facilities may report lower rates, the pooled rates from facilities with higher rates could influence the overall findings. Also, geographical location variations introduce diverse healthcare infrastructures, cultural norms, and resource accessibility. These contextual disparities can distinctly shape healthcare delivery and patient engagement practices, potentially impacting the observed rates of LTFU.

We observed a high risk of loss to follow-up (LTFU) among HIV registrants in the initial 24 months of follow-up, followed by a decline until 36 months, a rise at 48 months, and a subsequent decline until the end of 60 months. Our explanation for this observation is that when individuals start a new medication like antiretroviral therapy (ART), they might experience side effects and find it challenging to adapt to this new routine. Additionally, some individuals might feel stigmatized or embarrassed to attend their medication appointments, leading them to miss their schedules. With proper counseling and encouragement during this phase, registrants may adapt to their new routine, which may have improved their retention in care, contributing to the decline in LTFU observed from 12 to 36 months of follow-up. However, the gradual increase in LTFU cases observed again after 48 months of follow-up could be attributed to feelings of being burdened or tired, or experiencing long-term side effects from ART, which can cause registrants to drop out of treatment. With ongoing support and encouragement from healthcare providers, as well as assessment and management of drug side effects, registrants may adapt and continue care, possibly explaining the decline observed after 48 months. This highlights the importance of closely monitoring individuals on long-term ART to assess and address the risk of treatment disengagement.

Our findings revealed an increase in LTFU cases from 2019 to 2020, followed by a decline, except for registrants ≤ 24 years and males, who exhibited an upward trajectory from 2021 to 2022. The increase in cases from 2019 to 2020 could be attributed to the COVID-19 pandemic, which disrupted ART services globally and in Ghana (48, 49). During this period, restrictions on movement, healthcare resource reallocation, and fear of infection may have contributed to the interruption of ART services. The observed decline after this period likely reflects the restoration and recovery of ART services to normal operations post-pandemic. However, the upward trajectory observed in the younger population and males from 2021 to 2022 suggests a demographic vulnerability that warrants specific attention. Younger individuals and males might face unique barriers, such as higher mobility, employment-related challenges, and social stigma, which can impact their adherence to ART. Addressing these issues through targeted interventions and support programs is crucial to improve retention in care for these vulnerable groups.

Our findings indicate that registrants with a high viral load of 1,000 copies or more are at higher risk of loss to follow-up (LTFU) compared to those with a viral load of less than 1,000 copies. This aligns well with studies conducted in Tanzania (30) and Korea (21), which also reported similar results. Individuals with higher viral loads might experience more severe health conditions, impacting their ability to adhere to treatment regimens (50). Additionally, psychological factors, including the perception of being at a more advanced stage of the disease due to a higher viral load, might affect patient motivation and engagement with treatment. The need for more intensive treatment strategies for higher viral loads could also pose logistical challenges, such as increased frequency of clinic visits and more complex medication regimens, leading to potential disengagement from care. These findings underscore the importance of targeted interventions to support individuals with high viral loads, including enhanced counseling, mental health support, and tailored treatment plans to improve adherence and retention in care.

We observed that registrants who adhered to antiretroviral therapy (ART) had a lower risk of loss to follow-up (LTFU) compared to those who did not adhere. These findings compare well with findings from studies in Tanzania and Ethiopia (30, 51). Registrants who adhere to ART may signify regular healthcare engagement, ensuring ongoing monitoring and support from healthcare providers. This engagement likely leads to better disease management, improved health outcomes, and reduced chances of disengagement from care. Furthermore, adherence to ART not only controls viral replication but also contributes to improved overall health, potentially motivating individuals to remain committed to their treatment regimen and clinic appointments. Consistent adherence to ART is crucial as it enhances the effectiveness of the treatment, prevents the development of drug resistance, and improves the quality of life for patients. These benefits underscore the importance of developing and implementing strategies to promote ART adherence among HIV registrants to ensure sustained engagement in care.

Our study found that registrants who disclosed their HIV status had reduced risk of LTFU compared to those who did not disclose their status. This aligns with findings from two studies in Ghana and Ethiopia (16, 51). Disclosure of one’s HIV status might play a pivotal role in enhancing engagement and retention in HIV care programs. The reason could be that there is improved social support and access to tailored healthcare services for individuals who disclose their HIV status. Disclosure might lead to increased understanding and acceptance within social circles, reducing stigma and facilitating access to psychological and emotional support. Moreover, disclosure could enable healthcare providers to offer more personalized care, including targeted counseling and assistance in treatment adherence.

This current study also found that registrants in WHO-defined HIV stage 2 and stage 3 had a reduced risk of loss to follow-up (LTFU) compared to those in stage 1. The increased severity of symptoms and potential complications associated with advanced disease stages (52, 53) may motivate individuals to engage more consistently in their healthcare. The fear of deteriorating health and the intensity of experiencing signs and symptoms of HIV infection could serve as significant motivating factors for regular clinic attendance and adherence to treatment regimens. Additionally, healthcare providers may prioritize monitoring and intervention for individuals in more advanced stages, potentially enhancing retention in care.

Our study, like all research, had a limitation that should be considered when interpreting these findings. Our study relied exclusively on secondary data sources, limiting the inclusion of potentially significant predictors such as body mass index and specific comorbidities like hypertension and diabetes. Despite this, our study demonstrates significant trends and predictors of LTFU in a rural context using paper-based medical records alone after employing a rigorous data extraction procedure.

## Conclusion

In this study, we investigated the incidence, trends, and predictors of LTFU among HIV registrants at Nandom District Hospital over a 5-year period (2018–2022). The study revealed an incidence proportion of 24.14% and a rate of 9.57 per 1,000 person-months. LTFU risk peaked in the initial 12 months and again between 36 and 48 months of follow-up, followed by a decline until 60 months. Overall, there was a decreasing trend in LTFU from 2018 to 2022, although individuals under 25 years and males exhibited a concerning increase in LTFU risk from 2021 to 2022. A notable predictor of increased LTFU risk was a viral load of 1,000 copies or more compared to less than 1,000 copies. Conversely, registrants in WHO-defined HIV stage 2 and 3, those adhering to medication, and those who disclosed their HIV status were less likely to experience LTFU. These findings contribute to the existing literature on LTFU and underscore the critical roles of clinical and sociodemographic factors in retention in HIV care.

## Data Availability

The original contributions presented in the study are included in the article/Supplementary material, further inquiries can be directed to the corresponding author.
