# Supplementary figures and images for: Predictors of loss to follow-up among people living with HIV on antiretroviral therapy in a rural health facility using paper-based records
Source: Front Public Health. 2025 Aug 21;13:1623805. doi: 10.3389/fpubh.2025.1623805 (PMC12408609; doi:10.3389/fpubh.2025.1623805)

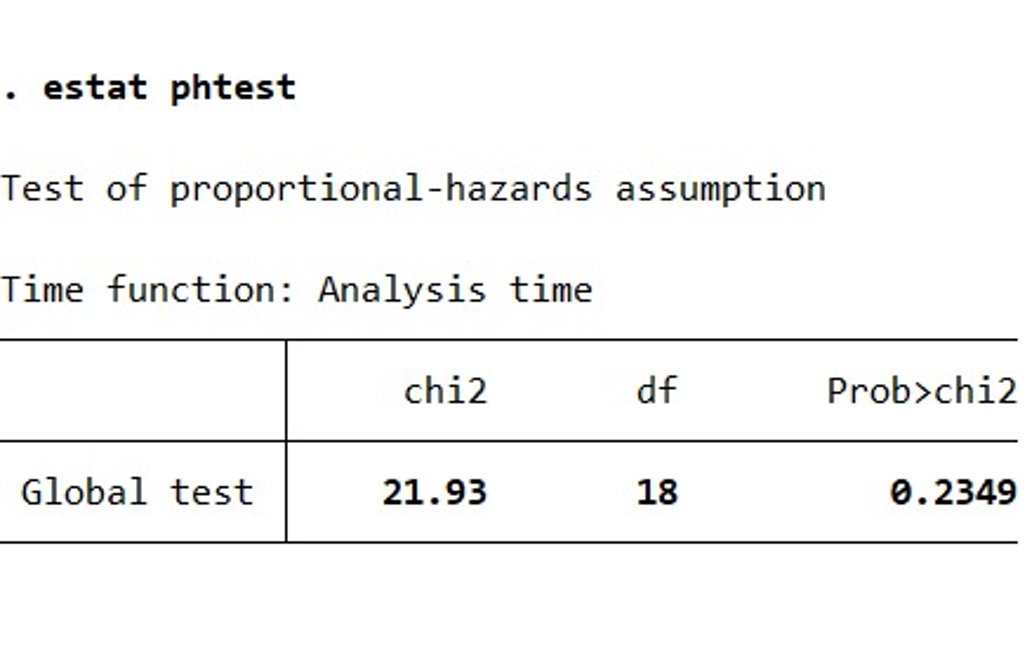

Supplement: Supplementary file 1 [file Image_1.TIFF]

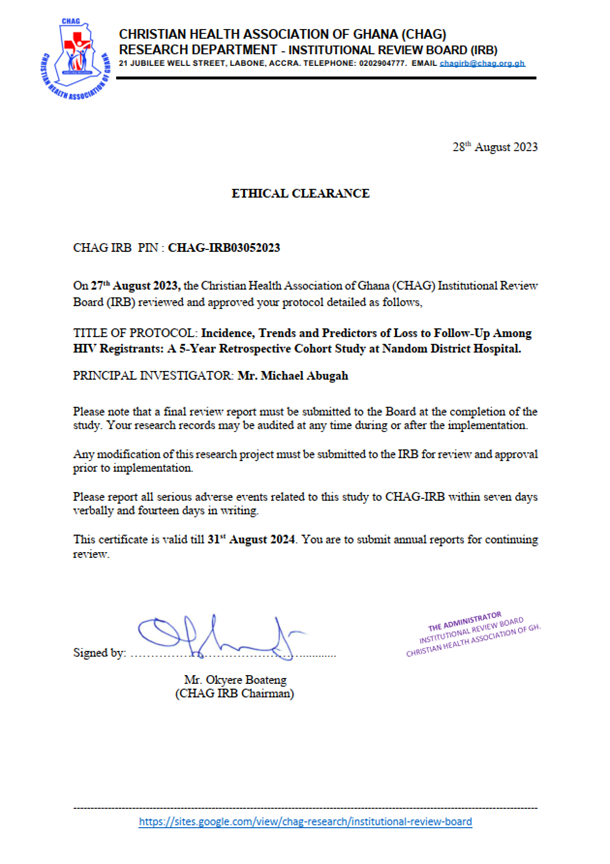

Supplement: Supplementary file 2 [file Image_2.TIFF]
